# Purkinje Cardiomyocytes of the Adult Ventricular Conduction System Are Highly Diploid but Not Uniquely Regenerative

**DOI:** 10.3390/jcdd10040161

**Published:** 2023-04-07

**Authors:** Hirofumi Watanabe, Ge Tao, Peiheng Gan, Baylee C. Westbury, Kristie D. Cox, Kelsey Tjen, Ruolan Song, Glenn I. Fishman, Takako Makita, Henry M. Sucov

**Affiliations:** 1 Department of Regenerative Medicine and Cell Biology, Medical University of South Carolina, Charleston, SC 29425, USA; 2 Leon H. Charney Division of Cardiology, New York University Grossman School of Medicine, New York, NY 10016, USA; 3 Darby Children’s Research Institute, Department of Pediatrics, Medical University of South Carolina, Charleston, SC 29425, USA; 4 Department of Medicine, Division of Cardiology, Medical University of South Carolina, Charleston, SC 29425, USA

**Keywords:** polyploidy, diploid cardiomyocyte, heart regeneration, conduction system, Purkinje cell

## Abstract

Adult hearts are characterized by inefficient regeneration after injury, thus, the features that support or prevent cardiomyocyte (CM) proliferation are important to clarify. Diploid CMs are a candidate cell type that may have unique proliferative and regenerative competence, but no molecular markers are yet known that selectively identify all or subpopulations of diploid CMs. Here, using the conduction system expression marker Cntn2-GFP and the conduction system lineage marker Etv1Cre^ERT2^, we demonstrate that Purkinje CMs that comprise the adult ventricular conduction system are disproportionately diploid (33%, vs. 4% of bulk ventricular CMs). These, however, represent only a small proportion (3%) of the total diploid CM population. Using EdU incorporation during the first postnatal week, we demonstrate that bulk diploid CMs found in the later heart enter and complete the cell cycle during the neonatal period. In contrast, a significant fraction of conduction CMs persist as diploid cells from fetal life and avoid neonatal cell cycle activity. Despite their high degree of diploidy, the Purkinje lineage had no enhanced competence to support regeneration after adult heart infarction.

## 1. Introduction

Cardiomyocytes (CMs) of the adult mammalian heart are understood to have very limited proliferative ability. In the aftermath of ventricular injury (e.g., a myocardial infarction) and the consequent loss of myocardium by cell death, the inability to appreciably regenerate lost CMs leads to permanently impaired cardiac output and a substantial risk of further progression to heart failure.

From an early view that the adult mammalian ventricle is completely nonproliferative, several lines of evidence now demonstrate that CM proliferation does occur. This has been seen in the context of CM turnover and replacement in the normal adult heart and as regeneration following adult heart injury [1,2,3]. However, the level of de novo generation of new CMs in both contexts is typically very low. This contrasts with highly efficient adult heart regeneration in zebrafish and some other nonmammalian vertebrates, and with highly efficient heart regeneration in mammalian late gestation embryos and early neonates [4]. In all such cases, including in the adult mammal, new CMs arise from preexisting CMs and not from a progenitor or other non-CM cell type [3,5]. Thus, mammalian CMs transition in the neonatal period from the state of high proliferative competence that characterizes the embryonic heart to a minimally proliferative and minimally regenerative state that persists through adulthood. In mice and rats, this transition occurs midway during the first postnatal week [6,7].

Concurrent with the neonatal transition in CM regenerative capacity is a change in CM ploidy. Like most cells of the body, embryonic CMs are diploid (one nucleus containing a 2n number of chromosomes, designated 1 × 2n, where n is the haploid genome). In mouse studies, it was found that during the first postnatal week, there is a burst of CM cell cycle activity that includes S-phase DNA replication but followed in most cases by interruption prior to completion of cell cycle, resulting in CMs with 4-times the haploid DNA level [8,9,10]. Depending on whether mitotic interruption occurs before karyokinesis or before cytokinesis and abscission, the resulting cell can have one 4n nucleus (designated 1 × 4n) or two 2n nuclei (designated 2 × 2n), respectively. Mice have an overwhelming majority of 2 × 2n CMs, but both polyploid CM types exist together in the later neonatal heart and throughout adulthood. To a lesser extent, subsequent rounds of cell cycle entry with mitotic interruption later in the first and second postnatal weeks result in some CMs with higher numbers of nuclei, higher numbers of genomes per nucleus, or both together. In mice and most other well-studied mammals, only a few percent of adult ventricular CMs are diploid. This transition to polyploidy does not occur in the highly regenerative zebrafish heart, which remains almost completely diploid throughout life [4]. Thus, across species and life stages, there is correspondence between the presence of a high level of diploid CMs and efficient proliferative capacity, and between a low level of diploid CMs and poor proliferative ability.

Several independent experimental approaches support the premise that the relation between CM ploidy and CM proliferation is not merely correlative but causative [9]. In zebrafish, genetic manipulations that led to a high level of polyploid CMs blocked regeneration after later adult heart injury [11,12]. Similarly, in mouse studies, forcing premature CM binucleation by genetic means in middle or late gestation reduced the number of CMs at birth and led to heart defects [13,14]. In our prior work [12], we demonstrated that the percentage of diploid CMs in the adult mouse ventricle is not a fixed number but rather is a variable trait subject to the combined influence of multiple natural genetic polymorphisms. Thus, some inbred mouse strains have a much higher level of diploid CMs compared to others, and we showed that mouse strains with more diploid CMs have better regeneration after adult injury. Although this relation is still controversial [15], in many adult mouse genetic studies a correlation has also been observed between a higher mononuclear CM level and higher CM proliferation [16,17,18,19,20].

It is likely that the adult diploid CM population is itself heterogeneous, with perhaps only a subset of these capable of supporting regeneration. A significant limitation in studying diploid CMs is the absence of molecular markers that define this CM population or its subcomponents. Even when comparing diploid to polyploid CMs, other than having only one diploid nucleus and generally being smaller than polyploid CMs, there is as yet no obvious distinction between these. Indeed, several bulk or single cell RNA-Seq attempts to identify diploid vs. polyploid CM gene expression differences were either unsuccessful or resulted in uncertain outcomes [21,22,23,24].

In this study, we evaluated the ploidy and regenerative capacity of the adult ventricular conduction system lineage. Purkinje myocytes are the terminal component of the cardiac conduction system and interface in the ventricular myocardium with so-called “working” CMs, or alternatively with transitional CMs that then interface with working CMs. Purkinje CMs (as well as other components of the conduction system) are derived from the CM lineage and share many molecular features with working CMs, including the expression of sarcomere proteins [25]; they are therefore commonly described as specialized CMs. The ploidy status of this CM subpopulation has not previously been addressed. We find that the adult conduction sublineage is remarkably highly diploid.

## 2. Materials and Methods

Mice. The BAC-transgenic Cntn2-GFP line was originally sourced from MMRRC (stock 029940-UCD) and obtained from Glenn Fishman (NYU, New York City, NY, USA). The Etv1Cre^ERT2^ knock-in line was obtained directly from JAX (strain 013048). The conditional R26-tdTomato line was obtained from JAX (strain 007914). All three alleles were outcrossed to a variety of strain backgrounds before this study began. To induce recombination in Etv1Cre^ERT2^/R26-tdT mice, tamoxifen (T5648, Sigma-Aldrich, St. Louis, MO, USA; 100 mg/kg body weight in corn oil) was administered by i.p. injection once per day for 5 days, with subsequent analysis no earlier than 48 hr after the last injection. All mice used for experimental purposes in this study were adults, generally of 3–6 months of age, and both sexes were used with no sex-based differences noted.

Whole-mount fluorescence. Using fresh isolated hearts, an incision was first made along the long axis of the anterior surface of the left ventricle to expose the ventricular septal surface and the left ventricular free wall. The right ventricle was then exposed through an incision along the long axis of the anterior surface of the right ventricle. Each interior surface was photographed by fluorescence microscopy (Keyence BZ-X810) using the automatic image stitching function.

Ventricular single cell preparations. As previously described [12,26], hearts were removed from anesthetized heparin-treated mice and immersed in ice-cold Kruftbrühe (KB) solution (70 mM potassium aspartate, 40 mM KCl, 15 mM KH_2_PO_4_, 10 mM glucose, 10 mM taurine, 0.5 mM EGTA, 10 mM sodium pyruvate, 10 mM HEPES, 5 mM BDM, 0.5% BSA) to stop contraction. In a typical procedure, hearts were quickly attached to a Langendorff perfusion rig, retrograde perfused with calcium-free Tyrode’s solution (120 mM NaCl, 4 mM KCl, 0.33 mM NaH_2_PO_4_, 1 mM MgCl_2_, 10 mM HEPES, 11 mM glucose, 20 mM taurine, 20 mM BDM), and then digested with 50 mg collagenase type II (1 mg/mL) in calcium-free Tyrode’s solution. In some cases, following heart dissection and immersion in KB solution, hearts were digested by anterograde perfusion [27] using 0.25 mg/mL Liberase (05401127001, Roche, Basel, Switzerland) in calcium-free Tyrode’s solution, which modestly increased the yield of conduction cells. Ventricular tissue alone (the lower half of the ventricle) was manually removed using forceps and then triturated by gentle pipetting in KB solution to make a cell suspension. This was filtered by gravity through a 250 μm nylon mesh and then fixed in 2% paraformaldehyde (PFA) in PBS at room temperature for 15 min. To facilitate increased recovery and isolation of larger clumps of tissue, a larger 400 μm mesh screen was used. Staining was performed with the following primary antibodies: rabbit anti-RFP (tdT) (200-301-379; Rockland, Pottstown, PA, USA, at 1:200); chicken anti-GFP (ab13970; Abcam, Cambridge, UK, at 1:500); and goat anti-connexin40 (sc−20466, Santa Cruz, CA, USA, at 1:250). AlexaFluor-coupled secondary antibodies were all obtained from Thermo Fisher Scientific.

Nuclear number and ploidy. Numbers of nuclei per cardiomyocyte and their ploidy were quantified using photographs taken at a uniform setting for all cell preparations in an experiment with a Leica DFC3000G camera in full frame mode (1296 × 966 pixels) on an Olympus BX41 microscope (20× objective), or with a Keyence BZ-X810 with a built-in camera (1920 × 1440 pixels). Mononuclear percentage was defined by counting at least 100 cells per slide from at least five slides per heart preparation. Nuclear ploidy of cardiomyocytes was measured based on DAPI or Hoechst fluorescence intensity in images, calculated using ImageJ, and normalized to the median intensity value of binucleated CM nuclei (defined as diploid).

Statistical analysis. ANOVA with Tukey-Kramer correction was used to calculate statistical values of significance using the program JMP Pro (Version 17) (SAS Institute, Inc.; Cary, NC, USA).

Infarction and analysis. Tamoxifen was administered to Etv1Cre^ERT2^/R26-tdT mice by i.p. injection once per day for 5 days, and the day after the last dose, mice were subjected to left anterior descending artery (LAD) ligation. After LAD ligation, an Alzet pump (model 1002, release rate 0.25 µL/hr, 14-day minimum capacity; Durect Corp., Cupertino, CA, USA) containing 50 mg/mL EdU (A10044, Thermo Fisher Scientific, Waltham, MA, USA; dissolved in DMSO then diluted 1:1 with saline) was implanted subcutaneously under the back skin. Then, 14 days later, the Alzet pumps were removed under light anesthesia, and 2 days later the mice were euthanized and hearts removed for analysis. Noninjured (sham) mice were siblings of the infarcted mice and were treated identically except without LAD ligation.

EdU analysis. Neonatal Cntn2-GFP mice were subcutaneously injected with 200 μg of EdU in 50 μL (consisting of 2 μL of a stock solution in DMSO diluted with 48 μL PBS) once daily from P2 to P5. Isolation of cardiomyocytes was performed at P32, with EdU and GFP staining as described below and above. Overall, 60 nonoverlapping images were taken at 20× magnification and the number of nuclei and ploidy of all cardiomyocytes evaluated as above. In P32 uninjured or adult infarcted hearts, EdU was visualized in cell suspensions (prepared as described above) using a Click-iT EdU AlexaFluor 594 Imaging Kit (C10339, Thermo Fisher Scientific, Waltham, MA, USA). When used, cell preparations were immunostained with mouse anti-cardiac troponin T antibody (ab8295, Abcam, Cambridge, UK, at 1:500), subsequently with AlexaFluor-488 conjugated goat anti-mouse IgG secondary antibody (A11001, Thermo Fisher Scientific, Waltham, MA, USA), to define CMs; in other cases CMs were identified by their natural autofluorescence in the green channel. Nuclei were stained with 5 μg/mL Hoechst 33342 (H3570, Thermo Fisher Scientific, Waltham, MA, USA) or 5 μg/mL DAPI for 5 min. Cells were washed in PBS then applied to microscope slides and mounted with ProLong Gold Antifade Mountant (P36930, Thermo Fisher Scientific, Waltham, MA, USA). Quantification of EdU signal was performed exactly as described above for DAPI and Hoechst signal measurement, except without normalization to any standard.

Histology. Freshly isolated hearts were immersed in ice cold KB buffer to arrest in diastole and fixed in 4% paraformaldehyde (PFA) in PBS at 4 °C overnight with gentle agitation on a rocker.

Hearts were dehydrated through increasing ethanol concentrations then embedded in paraffin; 5 μm sections were obtained with a Leica RM 2135 microtome. Antigen retrieval was performed in a microwave oven for 25 min in a pressure cooker using citrate antigen retrieval buffer (10 mM citrate, 0.05% Tween 20, pH 6.0). For EdU detection, Click-iT EdU AlexaFluor 594 Imaging Kit (C10339, Thermo Fisher Scientific, Waltham, MA, USA) was used following the product instructions. After subsequent blocking with 5% blotting grade milk (1706404, Bio-Rad, Hercules, CA, USA) for 2 hr, heart tissue sections were incubated at 4 °C overnight with primary antibodies: mouse anti-cardiac troponin T antibody [1C11] 1:500 (ab8295; Abcam, Cambridge, UK); rabbit anti-RFP at 1:200 (200-301-379; Rockland, Pottstown, PA, USA). On the following day, heart tissue sections were incubated at room temperature for 2 hr with secondary antibodies: goat anti-mouse IgG (H + L) cross-adsorbed secondary antibody AlexaFluor 488 conjugate 1:500 (A11001, Thermo Fisher Scientific, Waltham, MA, USA); goat anti-rabbit IgG (H + L) cross-adsorbed secondary antibody AlexaFluor 647 conjugate 1:500 (A21244, Thermo Fisher Scientific, Waltham, MA, USA). Intestines were removed freshly, formed into Swiss rolls [28], and fixed overnight in 10% formalin. The intestines were embedded in paraffin and cut into 5µm sections in the same manner as above. After deparaffinization, Edu detection was performed as above. Subsequently, nuclear staining was performed with 5 μg/mL Hoechst 33342 (H3570, Thermo Fisher Scientific, Waltham, MA, USA) for 15 min.

## 3. Results

### 3.1. An Unusually High Percentage of Purkinje CMs Are Diploid

**Figure 1:**
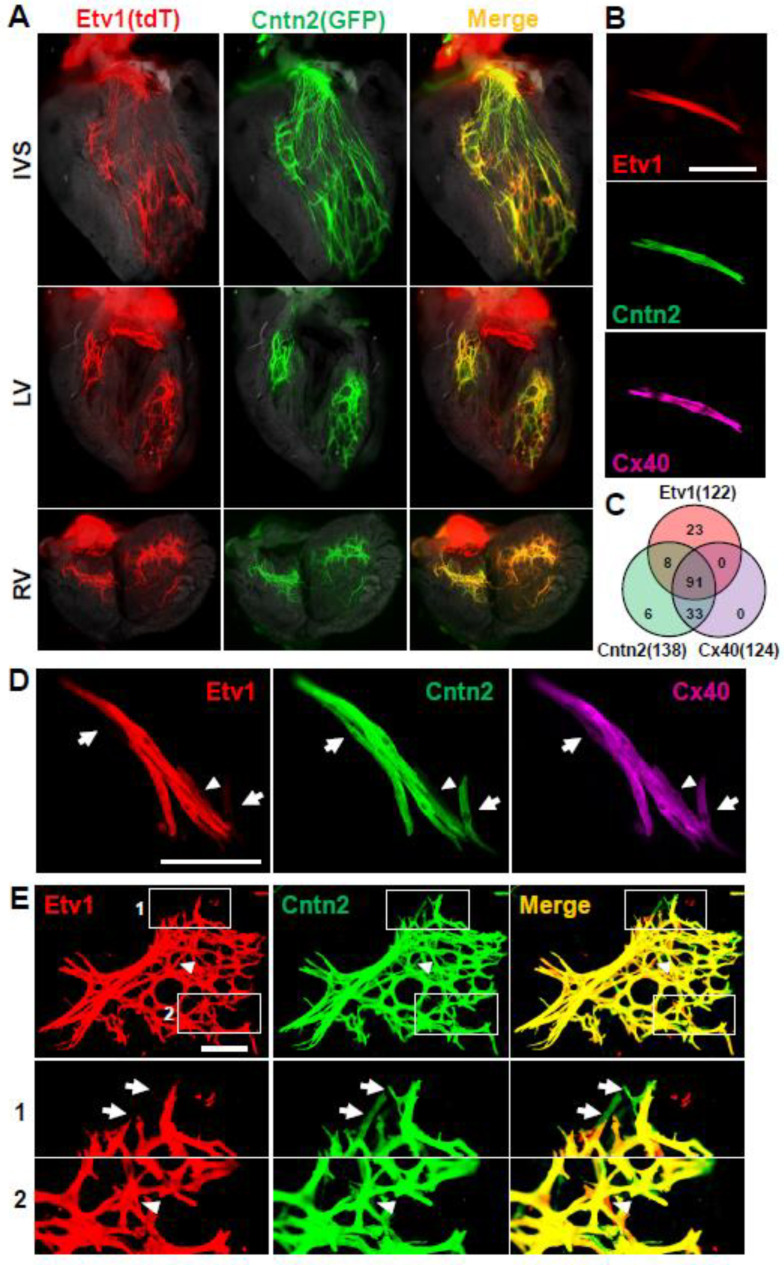
Heterogeneity of conduction system markers. (**A**) In situ views of the adult ventricular conduction system labeled by Etv1Cre^ERT2^/R26-tdT and Cntn2-GFP in the same mice. Unfixed hearts were dissected to expose the interventricular septum (IVS), left ventricle (LV), and right ventricle (RV) luminal surfaces. (**B**) An example of an isolated single cell that is triple labeled by tdT (Etv1), GFP (Cntn2), and Cx40. (**C**) Summary of overlap in expression of these three markers in 161 cells expressing at least one of these. Note that although there were no Cx40+ only or Cx40+Etv1+ double positive cells in this analysis, in other cell preps we observed such cells (see panel 1D). (**D**) A small cluster of conduction cells, most of which are triple positive; arrows point to two cells that are Cntn2+Cx40+ only, and the arrowhead points to one cell that is Etv1+Cx40+ only. (**E**) A clump of partially digested tissue containing an array of conduction fibers and cells. This preparation was stained only for GFP and tdT. The boxed regions are shown at higher magnification in the panels below. Arrows and arrowheads point to cells that are Cntn2+ only and Etv1+ only, respectively. Scale bars are 100 µm in B and D, 250 µm in E.

To visualize the ventricular conduction system, we obtained Cntn2-GFP mice, in which GFP is driven by regulatory sequences of the contactin-2 gene in a BAC transgene. The expression of this transgene in the cardiac conduction system has been previously described [29]. We also obtained Etv1Cre^ERT2^ mice, which carry a tamoxifen-regulated Cre^ERT2^ cassette knocked into the Etv1 (previously called ER81) gene [30]. Etv1 protein is required for the formation and functionality of the conduction system, and the expression of Etv1-lacZ and Etv1-GFP alleles in the ventricle is limited to the conduction system [31,32]. Because the use of Etv1Cre^ERT2^ for the conduction system has not previously been described, we crossed this allele with Rosa26-tdTomato (R26-tdT), in which tdTomato (tdT) is conditionally expressed after Cre-mediated recombination and combined with Cntn2-GFP. We treated adult mice with tamoxifen to initiate R26-tdT recombination, and by fluorescence visualized GFP and tdT expression patterns on the luminal (endocardial) surfaces of the ventricle (the localization of conduction system fibers [25]). Both reporters showed a comparable and mostly overlapping expression pattern throughout the ventricle (Figure 1A).

To further evaluate their overlap, we prepared collagenase-digested cell suspensions of ventricular myocardium from tamoxifen-treated adult mice carrying both markers. Notably, in this and subsequent analyses, we utilized only the lower half of the ventricle. This includes the anterior wall of the left ventricle and the lower portions of the ventricular septum and right ventricle, but excludes the atria, atrioventricular valves and chordae tendineae, and the atrioventricular node and His bundle at the top of the septum. Within the conduction system, this preparation is therefore selective for the peripheral Purkinje cell component. With respect to injury and regeneration studies below, this preparation also includes the domains of myocardium that are impacted by left anterior descending coronary artery ligation in experimental infarction.

We immunostained single cell preparations for GFP and tdT and costained for connexin 40 (Gja5), a recognized additional marker of the conduction system [33]. We counted all CMs that expressed any of these three markers to allow quantitative evaluation (Figure 1B,C). The majority of CMs (57%) that expressed one marker expressed all three. At the same time, there was heterogeneity in marker expression, in that double-positive and single-positive cells were also observed. This was true also for Cx40; in the initial analysis (Figure 1C), all CMs that expressed Cx40 also expressed Cntn2 (GFP), although in subsequent preparations, we identified an occasional cell that was Cx40+ but not GFP+ (e.g., Figure 1D). It is not immediately obvious how to explain this heterogeneity. In single cell preparations (Figure 1B) or in small clusters of cells (Figure 1D), there was no obvious distinction in size or morphology between those cells that were triple positive and those that expressed only one or two markers. When isolation conditions were adjusted to allow visualization of large cell clusters (Figure 1E; here stained only for GFP and tdT), individual cells that were only tdT+ or only GFP+ appeared to be integrated into conduction fibers the same as tdT + GFP+ cells. A likely interpretation therefore is that none of these markers are expressed uniformly in every conduction CM. If there are functional differences between conduction CMs that express different subsets of these markers, it is not obvious from their morphology or distribution.

**Figure 2:**
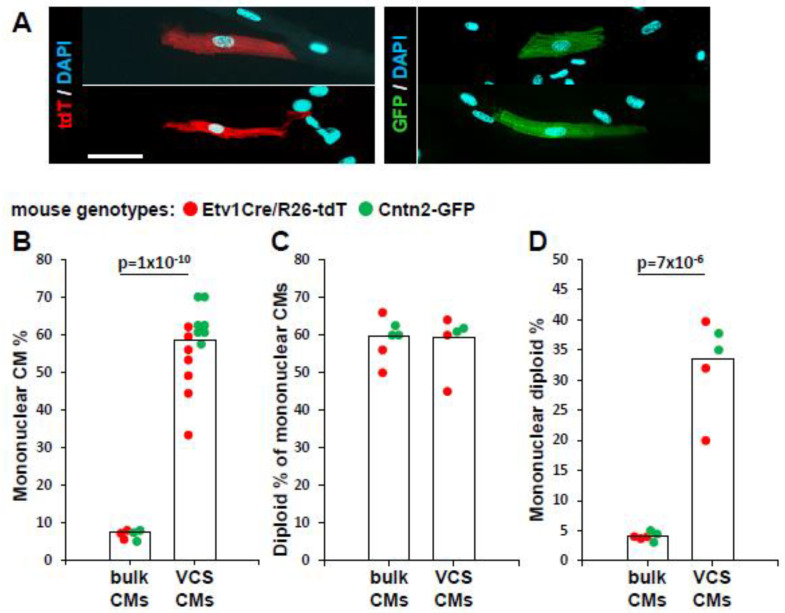
Purkinje CMs are highly diploid. (**A**) Examples of isolated mononuclear CMs of diverse morphology expressing either conduction system marker. Scale bar 100 µm and the same for all panels. It can be easily recognized that these cells are all mononuclear. (**B**–**D**) Ploidy analysis of bulk ventricular CMs (not expressing a conduction system marker) or of ventricular conduction system CMs (VCS CMs) expressing tdT (Etv1+) or GFP (Cntn2+) in single cell preparations. Each dot represents one mouse. Because there did not seem to be a substantial distinction in the analysis of the two lines, their data have been combined but color coded to indicate the original source. Some samples were scored for mononuclear CM% but not for nuclear ploidy.

As previously reported [34], Purkinje cells have a variety of morphologies. Conduction cells were typically long and slender (Figure 1B,D) although we also observed some that were more rectangular or block-like (Figure 2A). Based on similarity of appearance, the latter may be or may include transitional CMs, which for lack of a suitable molecular marker cannot be further confirmed.

In single-cell ventricular suspensions from mice separately expressing either Cntn2-GFP or Etv1Cre^ERT2^/R26-tdT individually, it was immediately obvious that the conduction cells were disproportionately mononuclear (Figure 2A). Mononuclear CMs comprised 7% of the overall ventricular CM population of these mixed-strain mice, a level typical for ventricular populations across many inbred mouse strains [12]. In contrast, over half of the Etv1 lineage (tdT+) or Cntn2-GFP+ CMs (57% for both combined) were mononuclear (Figure 2B). Because mononuclear CMs may have 2n or 4n (or higher) nuclei, we also measured the nuclear ploidy of the mononuclear CM population. Overall, 58% of the conduction mononuclear CM nuclei and 59% of the nonconduction (bulk) mononuclear CMs from the same hearts were 2n (the remainder being mostly 4n, with very few nuclei at higher ploidy level) (Figure 2C), which is typical for mice, as seen in our prior studies [12,35]. Thus, approximately one-third (57% × 58% = 33%) of the ventricular conduction CMs are 1 × 2n (diploid), compared to only 4% of bulk CMs (Figure 2D).

These ventricular single cell preparations typically yielded hundreds of thousands of CMs. In absolute terms, conduction CMs labeled by either Etv1Cre^ERT2^ (tdT+) or Cntn2-GFP represented only a small percentage (up to 0.3%, depending on preparation) of total ventricular CMs. After collagenase digestion, we observed that a fraction of conduction cells were present in clumps of undigested tissue (e.g., as in Figure 1E), perhaps because of a more elaborated matrix surrounding these fibers. In our usual single cell preparations, these clumps (also including incompletely digested bulk CMs) are excluded from analysis, but even with their inclusion, the number of conducting CMs is still quite low. Our estimate approaches the 1% level reported with a Sema3a-Cre^ERT2^ allele [36] using a similar cell isolation procedure. Because of their 8-fold higher degree of diploidy, conduction CMs therefore represent up to 3% (0.3% × 33%/4%) of the diploid CM population. Thus, even though the conduction population is itself highly diploid, it represents only a small fraction of the total diploid CMs of the ventricle.

### 3.2. Conduction CMs Avoid Cell Cycle to Remain Diploid

**Figure 3:**
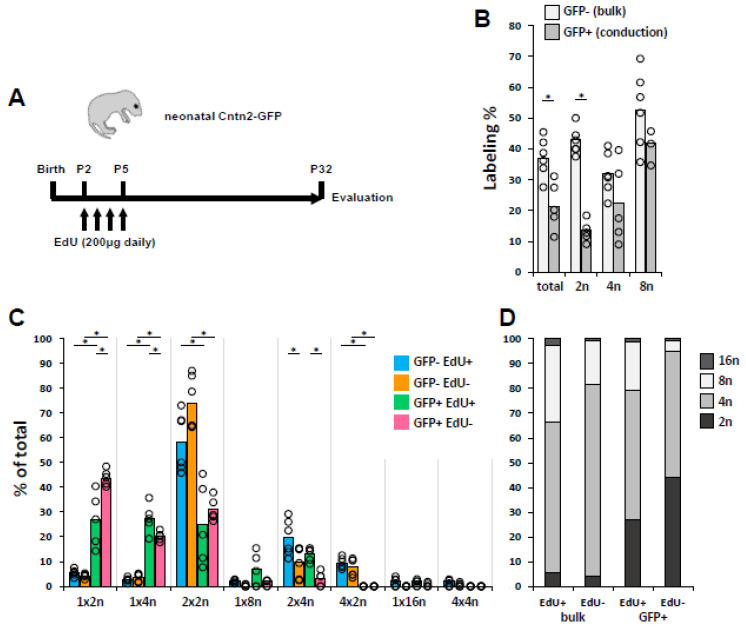
Cell cycle dynamics of bulk (nonconduction) and ventricular conduction (VCS) CMs based on neonatal EdU incorporation. (**A**) Schematic of the treatment protocol. (**B**) Percentage of nonconduction (GFP−) and conduction (GFP+) CMs that were labeled by EdU when analyzed at P32. Data for each are shown for all CMs (total) and then broken down by individual ploidy class. Each dot represents cells from one mouse. (**C**) Distribution of EdU+ and EdU− cells for GFP− (bulk) and GFP+ (conduction) CMs segregated by ploidy type. Each dot represents cells of the indicated type from one mouse. (**D**) Aggregation of the data in panel C by ploidy level. The 2n bars here are identical to the 1 × 2n bars in panel C, the 4n bars here are the sum of the 1 × 4n and 2 × 2n bars in panel C, etc. Statistical values for all comparisons in panels B and C are provided in Supplementary Materials; * indicates statistically significant differences.

Virtually all fetal CMs are diploid; CM polyploidy in mouse occurs during the first, and to a lesser extent, second postnatal week when CMs enter the cell cycle and replicate their DNA but fail to complete cytokinesis [8,9]. In principle, diploid CMs observed in adult mice may not have entered the cell cycle at all (i.e., persisted from embryonic stage) or may have entered and completed the cell cycle during this period (i.e., divided). This has not been resolved for the bulk CM population nor for the conduction lineage. To address this, we injected Cntn2-GFP neonatal pups with EdU around noon once per day during the postnatal P2–P5 interval, allowed these mice to age to day 32, and isolated single ventricular cells for analysis (Figure 3A). Although the kinetics of whole animal EdU labeling have not been reported (to our awareness), for BrdU, it is estimated that a single injection provides only 2 hr of labeling time in adult mice [37,38]. Thus, the EdU-negative CM group includes cells that did not have any cell cycle activity during the P2–P5 period, plus cells that were in S-phase during P2–P5 but at a time after injection, when the level of systemic EdU was too low to result in labeling.

We evaluated the data in two ways. First, we calculated the percentage of CMs that became labeled (Figure 3B). P2–P5 neonatal EdU treatment resulted in the labeling of 37.3 ± 6.3% of bulk (nonconduction) CMs overall when examined at P32 (Figure 3B). The labeling percentage for the individual 2n, 4n, and 8n subpopulations is also shown in Figure 3B. In this analysis, EdU labeling frequency was slightly higher for nonconduction diploid CMs (42.5% ± 4.4%) than for nonconduction 4n CMs (31.9% ± 6.9%), although this difference was not statistically significant (*p* = 0.14). For cells to become tetraploid (4n), they must have started as diploid, entered the cell cycle, and then been interrupted prior to cell cycle completion (excluding the small number of polyploidy events that occur by cell fusion [39]). In other words, tetraploidy necessitates S-phase DNA replication. Because the 2n and 4n subpopulations had the same labeling percentage, this indicates that diploid CMs in the later postnatal heart do not avoid the cell cycle in the P2–P5 neonate in order to remain diploid (if so, they would be disproportionately EdU−), but rather entered and completed the cell cycle. Because 8n cells go through two rounds of S-phase, whereas 4n cells go through one, it is expected that 8n CMs would have a higher labeling frequency as they have a higher likelihood of becoming labeled during at least one of these cycles over the four days of treatment.

We also calculated the relative contribution of different ploidy subtypes to the labeled and unlabeled populations (Figure 3C,D), as this gives a different view of the dynamics of these populations. Overall, diploid CMs accounted for 5.4% ± 1.4% of bulk EdU+ CMs and 4.3% ± 0.8% of bulk EdU− CMs, which was a nonsignificant difference (*p* = 0.12; Figure 3C,D). In contrast, relative to unlabeled CMs, the bulk EdU+ population included a smaller fraction of 4n CMs (61.0% ± 11.2% compared to 77.3% ± 9.1%; *p* = 0.020) but a larger fraction of both 8n (31.0% ± 9.7% vs. 17.8% ± 9.2%; *p* = 0.036) and 16n (2.6% ± 0.9% vs. 0.6% ± 0.7%; *p* = 0.002) cells (Figure 3D). This reflects the circumstance that CMs that transit S-phase more than once (in order to become 8n) during P2-P5 have a greater likelihood of becoming labeled.

When the GFP+ CMs from the same mice were analyzed, a much different profile emerged. Here, the labeling percentage overall was lower than the bulk CM population (21.7% ± 7.8% vs. 37.3% ± 6.3%; *p* = 0.005) and much lower for the diploid CM population (13.3% ± 3.4% vs. 42.5% ± 4.4%; *p* = 7 × 10^−6^) (Figure 3B). This indicates substantially lower S-phase activity for conduction CMs in the neonatal period and particularly for diploid conduction CMs. Consequently, diploid CMs were a higher fraction of the EdU− than of the EdU+ conduction cells (43.9% ± 3.3% vs. 26.8% ± 10.9%; *p* = 0.0005); Figure 3C,D. These observations indicate that a significant fraction of conduction CMs avoid cell cycle activity during the P2–P5 period to remain diploid. It is likely that these persist from fetal life as diploid cells and remain diploid throughout postnatal life, although an alternative is that they have cell cycle activity either before or after the P2–P5 period. The strikingly high overall diploid level of conduction CMs (Figure 1) was manifest in a much higher proportion of diploid CMs within both the EdU+ and EdU− subgroups relative to bulk CMs (Figure 3C,D). From these data, we cannot distinguish if the conduction CMs that do enter the cell cycle have an enhanced ability (relative to bulk CMs) to complete it, or simply do not undergo as many subsequent rounds of cell cycle with the probability at each to become polyploid.

In addition to quantifying the percentage of CMs that were EdU-labeled, we also measured the intensity of the EdU signal in 1 × 2n, 1 × 4n, and 2 × 2n CM nuclei (Supplementary Figure S1). Signal intensity ranged broadly, as expected because of two determinants: the variable EdU concentration when each cell was in active S-phase DNA replication, and the potential dilution or increase in signal if a previously labeled cell underwent an additional round of replication without or with further incorporation of more EdU. There was stronger labeling in the 1 × 2n and 2 × 2n bulk CM subgroups, on average approximately twice as much, compared to the conduction CMs. This is consistent with at least these subpopulations having been more active in cell cycle for bulk compared to conduction CMs. For 1 × 4n CMs, it was expected that these would on average have a higher EdU signal than 1 × 2n or 2 × 2n CMs because all of the incorporated EdU label is concentrated into one nucleus. This was true for the conduction 1 × 4n CMs but not for the bulk 1 × 4n CMs; a possible explanation might involve more of the latter proceeding into 8n and 16n ploidy classes. In sum, in terms of both percentage of EdU labeling and degree of EdU labeling, the analysis is consistent with the interpretation that conduction CMs are overall less active in cell cycle than bulk CMs.

Among bulk CMs, 4n CMs account for the largest subtype of CM, and of these, the vast majority are 2 × 2n rather than 1 × 4n (Figure 3C), as has been seen repeatedly in mouse and rat studies. In contrast, the 4n conduction subpopulation was roughly evenly divided between 1 × 4n and 2 × 2n cells (Figure 3C). This suggests a higher propensity of the conduction cells to arrest cell cycle progression prior to the completion of karyokinesis, rather than completing karyokinesis and then arresting in cytokinesis as occurs with most bulk CMs. At present, there is no explanation for why this occurs.

### 3.3. Ventricular Conduction System CMs Are Not Responsible for Heart Regeneration

For several reasons, we evaluated the possibility that the Purkinje cell lineage might be preferentially competent to support proliferation after adult myocardial infarction. First, diploid CMs are a candidate CM subtype that may have elevated regenerative ability, and as reported above, the conduction lineage is highly diploid. Second, lineage tracing revealed that proliferative CMs are preferentially located on the endocardial side of the ventricle [17], which is where conduction CMs are also located. Third, the very low level of Purkinje CMs (up to 0.3% of total CMs) measured above is consistent with the very low level of CM cell cycle activity in adult hearts, as measured by static markers or nucleotide incorporation.

**Figure 4:**
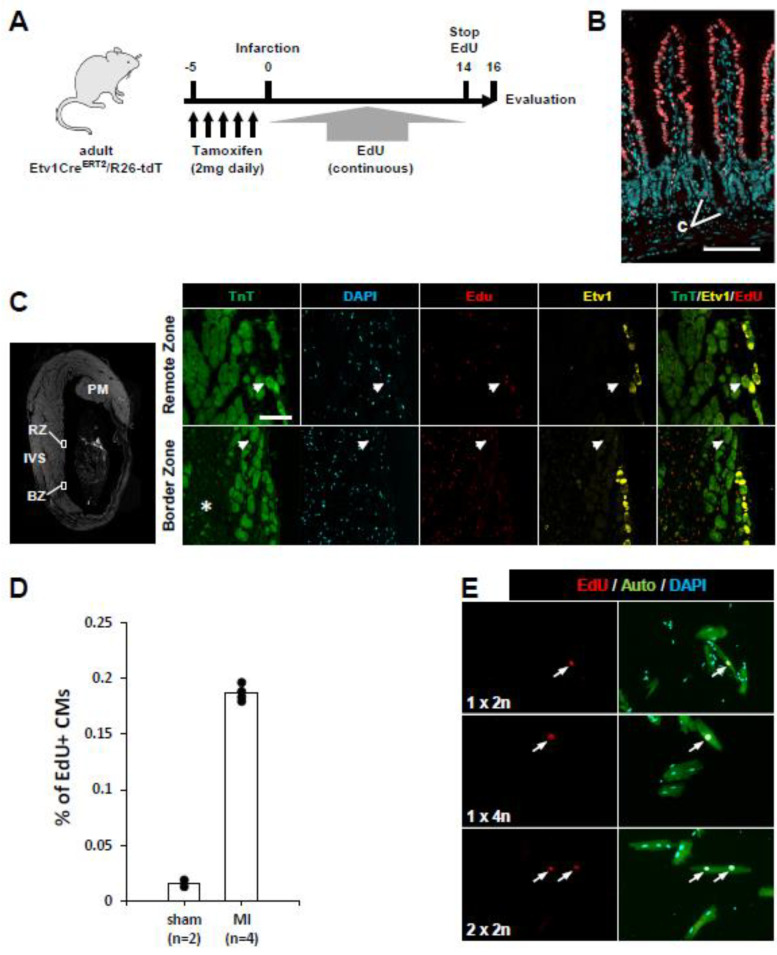
The Purkinje lineage does not contribute to heart regeneration. (**A**) Schematic of the protocol for infarction and EdU labeling. (**B**) Histology of the small intestine. Because of the 2-day period without label, the crypts (**C**) are unlabeled, whereas the villi are labeled from midway to tip, indicating the persistence of EdU throughout the labeling period. Scale bar 100 µ. (**C**) Histology of infarcted heart. The small boxes indicates the locations in the remote zone (RZ) myocardium and in the infarction-adjacent border zone (BZ), where the higher magnification views were obtained. PM, papillary muscle; IVS, interventricular septum. Arrows in the high magnification views point to EdU+ nonconduction CMs; the asterisk (*) is placed in the infarction region. Scale bar 100 µ. (**D**) Quantitation of EdU labeling in CMs from uninjured (sham) or infarcted (MI) adult mice, as analyzed in single cell preparations. (**E**) Examples of EdU+ CMs of the indicated ploidy classes in single cell preparations isolated from infarcted hearts. Arrows point to EdU+ nuclei. Auto, autofluorescence in the green channel (specific for CMs).

With a Cre-based lineage marker, it was possible to address this possibility. For this analysis, adult Etv1Cre^ERT2^/R26-tdT mice were treated with tamoxifen and then injured by permanent LAD coronary artery ligation (Figure 4A). We inserted an Alzet minipump at the time of infarction to release EdU, removed this after 14 days, and after 2 more days isolated hearts or heart cells for analysis. Histology of the intestine isolated at the same time confirmed the effectiveness of this EdU treatment protocol (Figure 4B). Sections of the recovered hearts revealed prominent infarctions across the left ventricular anterior wall and extending into the ventricular septum (Figure 4C). Staining in the infarction border zone revealed EdU labeling in numerous nonmyocytes and in occasional CMs (Figure 4C).

Because the ploidy of individual CMs cannot be determined in histology sections, we prepared ventricular single-cell suspensions from uninjured (sham) and infarcted hearts. In sham hearts, 0.016% ± 0.003% (n = 2) of CMs were labeled during this 2 week period (Figure 4D). This corresponds to an annual labeling rate of 0.4%. After injury, labeling was 11-fold higher (0.19% ± 0.01%; n = 4; Figure 4D), consistent with the observation that heart injury induces CM cell cycle activity. This is likely to be an underestimate of in vivo labeling because of the uncertain efficiency of collagenase digestion and cell recovery in the underperfused infarct-adjacent region where cell cycle activity is highest. EdU labeling is only an indication of a cell that has entered the cell cycle and progressed through S-phase DNA replication. However, if an EdU+ cell is mononuclear and has a diploid nucleus, it is assumed to have begun as a diploid cell, entered the cell cycle and replicated its DNA, and then completed cytokinesis (i.e., proliferated; see [40] for considerations related to this assumption). In this analysis of EdU+ single ventricular CMs after injury (Figure 4E), 54% ± 10% were mononuclear, and of these, 38% ± 18% had diploid nuclei, i.e., 20% of EdU+ labeled CMs were diploid (n = 4 hearts, 145 total EdU+ CM scored). This level is somewhat higher than the 11–13% estimate using genetic labeling methods [17,41] of adult CMs that complete cytokinesis (i.e., proliferate) after entering the cell cycle; the discrepancy could result from mouse strain background or overcounting or undercounting based on a variety of technical elements, etc. The remaining 80% of EdU incorporation events resulted in higher levels of ploidy. The conclusion from this aspect of the analysis is that cell cycle entry events followed by proliferation or followed by higher polyploidization were all readily observed in these infarcted mice.

Importantly, in these single-cell preparations, we did not identify any EdU+ and tdT+ double-positive cells. The recovery of tdT+ cells was equivalent in uninjured and injured hearts (both around 0.3%). Moreover, 219 tdT+ conduction CMs isolated from infarcted hearts were examined; if EdU incorporation occurred to an equal degree in conduction CMs as in bulk CMs (0.19%), we would expect less than one double-labeled cell within this 219 cell subset. Reciprocally, 145 EdU+ cells were examined; because the conduction lineage represents 0.3% of total ventricular CMs, we would likewise expect less than one double-labeled cell within this 145 cell subset. The absence of recovered EdU+tdT+ cells therefore does not indicate that the conduction lineage does not engage in the cell cycle after injury. However, these results do indicate that the ventricular conduction lineage does not have a unique ability to enter the cell cycle, let alone to proliferate, despite this population being so highly diploid.

## 4. Discussion

Inefficiency of regeneration in the adult ventricle underlies many of the pathologies that are associated with heart injury and disease. A better understanding of the relatively low frequency of regenerative events might allow the development of approaches that improve endogenous heart regeneration. To this end, ventricular diploid CMs have emerged as a candidate population that may have enhanced regenerative competence. There is to date no known molecular marker that identifies the diploid CM population or any subpopulation. A discovery in the present study is that the Purkinje conduction lineage, as labeled by two independent markers (Etv1Cre^ERT2^/R26-tdT and Cntn2-GFP), is remarkably highly diploid.

Our analysis of EdU incorporation during the neonatal period allowed us to address the dynamics of polyploidization of both the bulk CM and the conduction CM populations. This revealed that bulk diploid CMs seen in the juvenile period (here studied at P32) become EdUlabeled during the neonatal phase to the same extent as bulk polyploid CMs. Because the latter must have become polyploid by entering the cell cycle, we conclude that the diploid CMs seen at P32 also entered the cell cycle during the same neonatal period. Thus, for bulk diploid CMs, there is no indication for persistence from fetal life. Rather, diploid CMs seen later in life are diploid by having entered and completed the cell cycle.

In contrast, the same analysis demonstrated that a significant percentage of conduction CMs are diploid because they avoid neonatal cell cycle activity. This was evident in two parameters both shown in Figure 3: the lower degree (relative to bulk CMs) of EdU labeling of the conduction population overall and particularly so for diploid conduction CMs relative to diploid bulk CMs, and the difference in the representation of diploid CMs in the EdU+ to the EdU− conduction (GFP+) populations. From this analysis, it is not possible to define an absolute percentage of conduction cells that avoid the cell cycle, but 30% (the difference in labeling percentage between diploid bulk CMs and diploid conduction CMs; Figure 3B) is a plausible estimate. An open question is whether those conduction CMs that do enter the cell cycle are better able to complete it relative to bulk CMs. The observed higher level of EdU+ diploid conduction CMs compared to EdU+ diploid bulk CMs could be explained by a higher rate of cell cycle completion or by a lower rate of subsequent cell cycle activity, or a combination of the two; these cannot be resolved with the present data.

The Purkinje population is allocated from bulk CMs during fetal life [25] and so has already initiated a unique gene expression program and functional diversification prior to birth. Thus, it is not surprising that conduction CMs and bulk CMs might differ in their response to the neonatal circumstances that induce cell cycle entry and interruption. For the conduction CMs, these differences are manifest in a much higher level of diploidy, a lower percentage that reaches higher levels of polyploidy (8n, 16n), and a much higher frequency of 1 × 4n compared to 2 × 2n CMs. It is unclear whether any properties of the Purkinje cells are impacted by these parameters. Among working CMs, cell size correlates positively with ploidy, which is generally assumed to reflect the necessity of more genomes to support the transcriptional output needed to maintain a larger cell. However, this premise has not been experimentally validated for bulk CMs or for Purkinje cells. Furthermore, even though a relatively high fraction (33%) of the Purkinje lineage is diploid, still a majority of these cells are polyploid, which perhaps accounts to some extent for the range of shapes and sizes of these cells.

With a Cre-based lineage marker in hand, we investigated the relationship of the Etv1Cre^ERT2^ conduction lineage to regeneration. Our results provide a clear indication that the Purkinje lineage, despite being so highly diploid, has no special competence to support CM proliferation. If anything, the observation that the conduction lineage experiences diminished cell cycle activity in the normal neonatal heart (Figure 3) is consistent with its apparent lack of cell cycle activity after adult injury. This outcome does not contradict the premise that diploid CMs may be more highly regenerative than polyploid CMs, as the Purkinje lineage is only a very small subset of the total diploid CM population. A separate subpopulation, yet to be defined, may account for the enhanced regeneration that is thought to be associated with diploid CMs.

## Data Availability

The data presented in this study are available within the article and its supplementary materials.

## Supplementary Materials

The following supporting information can be downloaded at: https://www.mdpi.com/article/10.3390/jcdd10040161/s1, Supplemental Table S1: Table of *p* values of statistical significance for Figure 3B. Supplemental Table S2: Table of *p* values of statistical significance for Figure 3C. Supplemental Figure S1: Intensity of EdU signals by bulk and conduction CM ploidy class following neonatal EdU exposure, in arbitrary units; grey bars indicate average.
