# Supplementary material for: Purkinje Cardiomyocytes of the Adult Ventricular Conduction System Are Highly Diploid but Not Uniquely Regenerative
Source: J Cardiovasc Dev Dis. 2023 Apr 7;10(4):161. doi: 10.3390/jcdd10040161 (PMC10140853; doi:10.3390/jcdd10040161)
Supplement: Supplementary file 1 [file jcdd-10-00161-s001.zip › jcdd-2289270-supplementary.pdf]

Supplementary Materials for Watanabe et al.

Supplemental Table S1. Statistical values for comparisons made in Figure 3B, calculated using Student T-test. Background color in Table S1 and Table S2 indicate values of statistical significance.

		Total	2n	4n	8n
GFP-	GFP+	0.005	7.6E-7	0.148	0.157

Supplemental Table S2. Statistical values for comparisons made in Figure 3C, calculated using Tukey-Kramer correction for multiple comparisons.

		1x2n	1x4n	2x2n	1x8n	2x4n	4x2n
GFP-Edu+	GFP-Edu-	0.9833	0.9837	0.1327	0.8478	0.0272	0.4434
GFP+Edu-	GFP+Edu+	0.0005	0.0081	0.8453	0.0951	0.0438	1
GFP+Edu+	GFP-Edu+	2.1E-05	7.0E-10	0.0009	0.1160	0.1857	7.1E-06
GFP+Edu-	GFP-Edu-	2.6E-09	2.7E-07	5.4E-05	0.9437	0.1734	0.0001

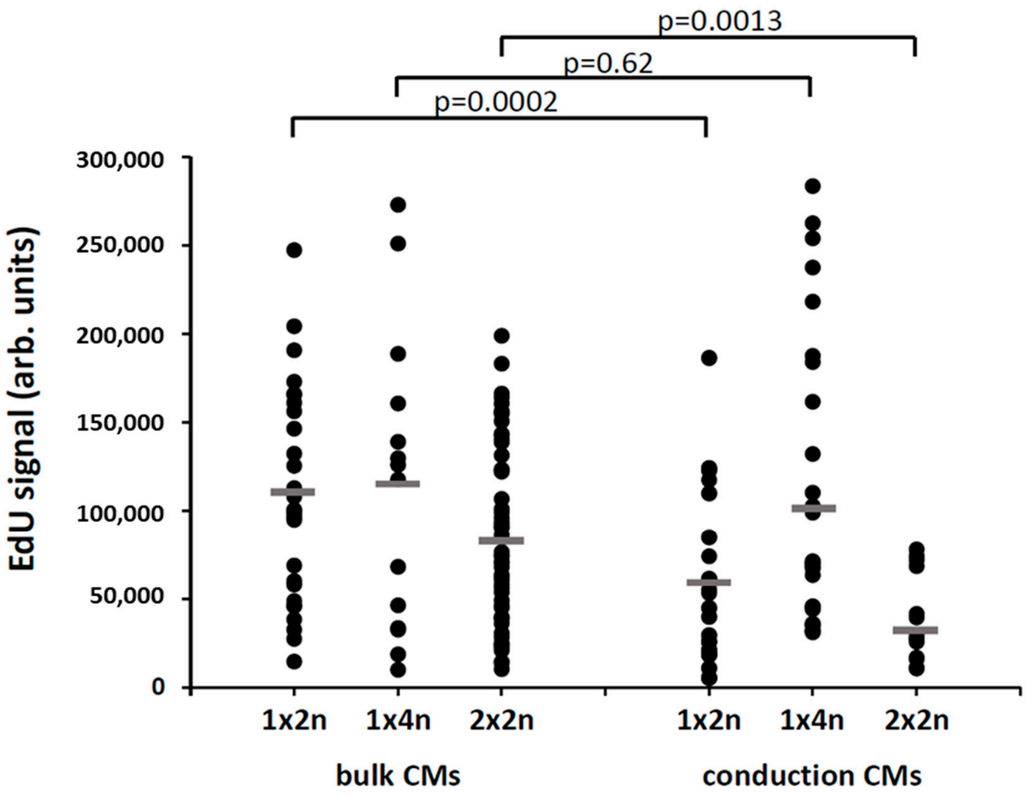

Supplementary Figure S1. Intensity of EdU signals by bulk and conduction CM ploidy class following neonatal EdU exposure, in arbitrary units; grey bars indicate average.
